# AI-ready rectal cancer MR imaging: a workflow for tumor detection and segmentation

**DOI:** 10.1186/s12880-025-01614-3

**Published:** 2025-03-14

**Authors:** Heather M. Selby, Yewon A. Son, Vipul R. Sheth, Todd H. Wagner, Erqi L. Pollom, Arden M. Morris

**Affiliations:** 1 S-SPIRE Center, Department of Surgery, Stanford University School of Medicine, Palo Alto, USA; 2 Department of Radiology, Stanford University School of Medicine, Palo Alto, USA; 3 Department of Radiation Oncology, Stanford University School of Medicine, Stanford, USA

**Keywords:** Magnetic resonance imaging, Rectal cancer, Segmentation, Artificial intelligence

## Abstract

**Background:**

Magnetic Resonance (MR) imaging is the preferred modality for staging in rectal cancer; however, despite its exceptional soft tissue contrast, segmenting rectal tumors on MR images remains challenging due to the overlapping appearance of tumor and normal tissues, variability in imaging parameters, and the inherent subjectivity of reader interpretation. For studies requiring accurate segmentation, reviews by multiple independent radiologists remain the gold standard, albeit at a substantial cost. The emergence of Artificial Intelligence (AI) offers promising solutions to semi- or fully-automatic segmentation, but the lack of publicly available, high-quality MR imaging datasets for rectal cancer remains a significant barrier to developing robust AI models.

**Objective:**

This study aimed to foster collaboration between a radiologist and two data scientists in the detection and segmentation of rectal tumors on T2- and diffusion-weighted MR images. By combining the radiologist's clinical expertise with the data scientists’ imaging analysis skills, we sought to establish a foundation for future AI-driven approaches that streamline rectal tumor detection and segmentation, and optimize workflow efficiency.

**Methods:**

A total of 37 patients with rectal cancer were included in this study. Through radiologist-led training, attendance at Stanford’s weekly Colorectal Cancer Multidisciplinary Tumor Board (CRC MDTB), and the use of radiologist annotations and clinical notes in Epic Electronic Health Records (EHR), data scientists learned how to detect and manually segment tumors on T2- and diffusion-weighted pre-treatment MR images. These segmentations were then reviewed and edited by a radiologist. The accuracy of the segmentations was evaluated using the Dice Similarity Coefficient (DSC) and Jaccard Index (JI), quantifying the overlap between the segmentations delineated by the data scientists and those edited by the radiologist.

**Results:**

With the help of radiologist annotations and radiology notes in Epic EHR, the data scientists successfully identified rectal tumors in Slicer v5.7.0 across all evaluated T2- and diffusion-weighted MR images. Through radiologist-led training and participation at Stanford’s weekly CRC MDTB, the data scientists’ rectal tumor segmentations exhibited strong agreement with the radiologist’s edits, achieving a mean DSC [95% CI] of 0.965 [0.939-0.992] and a mean JI [95% CI] of 0.943 [0.900, 0.985]. Discrepancies in segmentations were attributed to over- or under-segmentation, often incorporating surrounding structures such as the rectal wall and lumen.

**Conclusion:**

This study demonstrates the feasibility of generating high-quality labeled MR datasets through collaboration between a radiologist and two data scientists, which is essential for training AI models to automate tumor detection and segmentation in rectal cancer. By integrating expertise from radiology and data science, this approach has the potential to enhance AI model performance and transform clinical workflows in the future.

**Supplementary Information:**

The online version contains supplementary material available at 10.1186/s12880-025-01614-3.

## Introduction

Magnetic Resonance (MR) imaging is the optimal modality for diagnosing and staging rectal cancer due to its exceptional soft-tissue contrast, while also playing a critical role in assessing treatment response and detecting local recurrence [1]. Compared with computed tomography (CT), anatomical T2-weighted MR images have better soft tissue contrast, with potential for more accurate tumor segmentation in the pelvis by radiologists [2]. As a preliminary step in MR image interpretation, segmentation involves identifying and delineating rectal tumors on a representative 2D Region of Interest (ROI) or 3D Volume of Interest (VOI). Diffusion-weighted MR images enables  the visualization of the diffusion of water molecules in tissues which helps to distinguish tumors from normal soft tissue. The use of T2- and diffusion-weighted MR images for segmentation is now considered the standard of care for rectal cancer staging, treatment planning, and surveillance [3, 4]. The ability to accurately detect and segment tumors on MR images is critical for rectal cancer diagnosis and management, particularly as Artificial Intelligence (AI) applications increasingly rely on such segmentation for training and implementation.

Segmenting rectal tumors on MR images is prone to error [5]. The small size and indistinct boundaries of rectal tumors within the pelvis, along with their invasion of the rectal wall or nearby organs, make differentiation from normal tissue challenging. Tumors extending into the lumen may be mistaken for stool, further complicating segmentation [6]. Accurate segmentation often requires analysis of multiple MR imaging sequences, such as T2- and diffusion-weighted MR images, but software like Slicer v5.7.0 [7] lacks the functionality of picture archiving and communication system (PACS) workstations for simultaneous multi-parametric imaging review [8]. Additionally, inter- and intra-reader variability, influenced by tumor complexity and reader experience, contributes to errors in up to 40% of cases [2]. Segmentation inaccuracies can significantly impact treatment decisions. Suboptimal tumor delineation may lead to inadequate radiation targeting or unnecessary exposure of healthy tissues, reducing treatment efficacy or increasing side effects, such as damage to the bladder or bowel. Furthermore, errors in assessing response to neoadjuvant therapy may misguide decisions between a watch-and-wait approach and surgery, potentially compromising outcomes. These challenges underscore the need for standardized imaging protocols to enhance segmentation accuracy and consistency in clinical practice.

Segmentation of rectal tumors on MR images is a labor-intensive and time-consuming process requiring the expertise of fellowship-trained radiologists. The gold standard for research studies is independent review and segmentation by multiple radiologists, but this approach is often cost-prohibitive. A more feasible yet still demanding alternative involves a single radiologist performing all segmentation tasks, which can strain research budgets [9]. AI offers a promising solution for automating tumor segmentation, but unlike other cancers, publicly available MR datasets for rectal cancer are lacking [10]. Developing AI-ready datasets is essential for advancing multi-center research and clinical trials. These standardized datasets enable consistent data aggregation across institutions, fostering robust and generalizable AI models that account for variations in demographics, imaging protocols, and tumor presentations. They also streamline patient selection, biomarker identification, and real-time monitoring in clinical trials, accelerating discovery, reducing costs, and enhancing precision. To bridge the gap toward automatic segmentation, this study facilitated collaboration between radiologists and data scientists, enabling non-clinician data scientists, under expert radiologist oversight, to assist in the detection and segmentation of rectal tumors on T2- and diffusion-weighted MR images from 37 patients enrolled in a Phase II clinical trial.(NCT04380337) [11].

## Methods

**Fig. 1:**
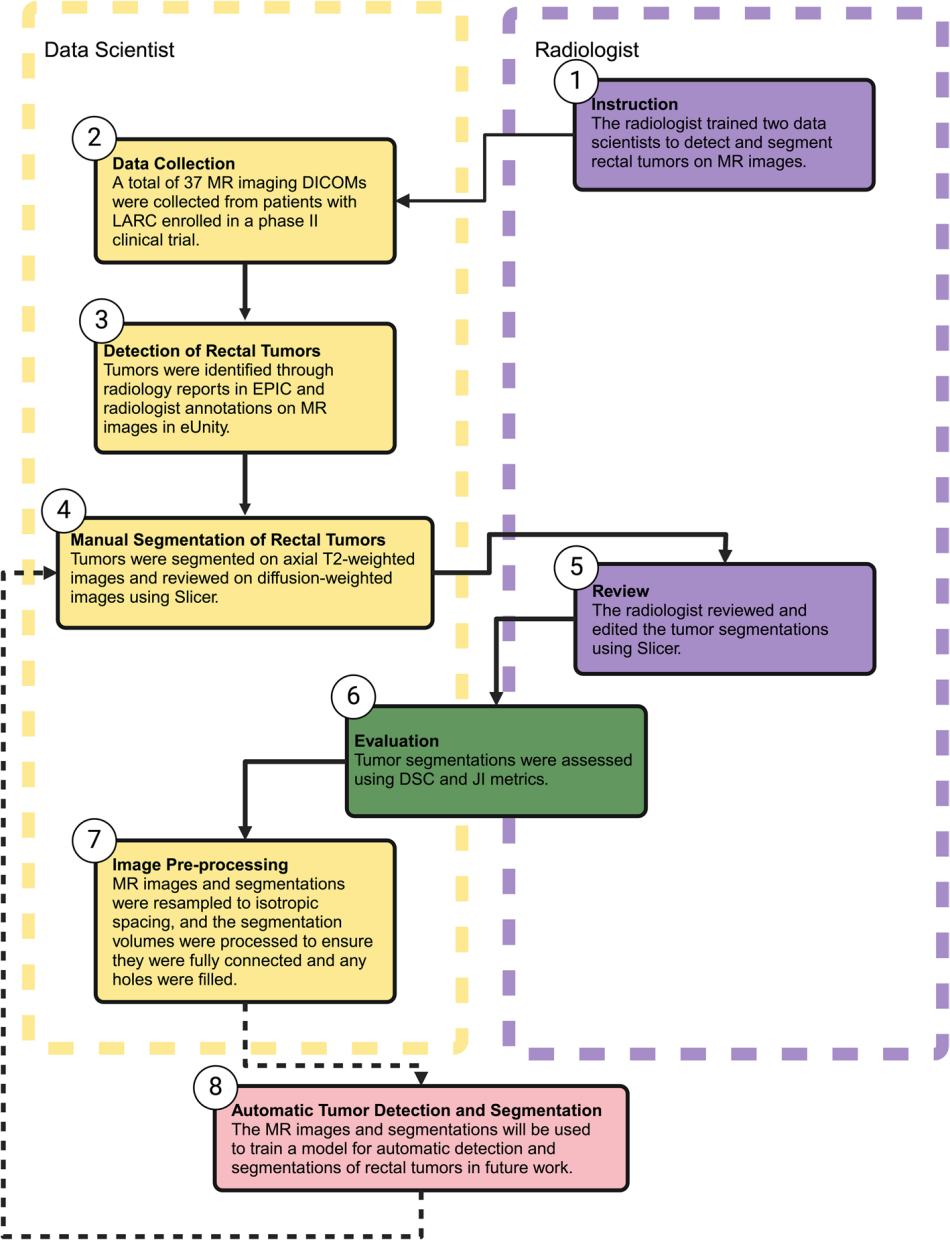
Workflow for the detection and manual segmentation of rectal tumors on MR image. Created in BioRender, Selby, H. (2025) https://BioRender.com/i64b822

The collaborative workflow between data scientists and radiologists for detecting and manually segmenting rectal tumors on MR images is illustrated in Fig. 1. This process includes the following steps: 1) instruction, 2) data collection, 3) detection of rectal tumors, 4) manual segmentation of rectal tumors, 5) review, 6) evaluation, and 7) image pre-processing.

### Instruction

Two data scientists attended weekly Colorectal Cancer Multidisciplinary Tumor Board (CRC MDTB) discussions to gain familiarity with rectal tumor detection and segmentation on MR images. At our institution’s CRC MDTB, the radiologist presents the MR images, and the pathologist presents the biopsy pathology, followed by discussions that also included surgeons, medical oncologists, radiation oncologists, and others.

The data scientists first received instruction from a radiologist about detecting rectal tumors on both T2- and diffusion-weighted MR images. Their training began with a hands-on mentorship phase, during which they spent three days shadowing the radiologist in the hospital to observe clinical workflows and tumor identification techniques. To develop their detection and segmentation skills, they practiced on 50 MR images from rectal cancer patients who were not included in this study, ensuring exposure to a variety of cases. These images were specifically selected based on the presence of rectal cancer and the absence of prior treatment, allowing for consistent training examples. This practice phase emphasized the importance of well-annotated MR images and the need for a standardized MR imaging protocol in the study. Beyond in-person training, the data scientists participated in numerous with the radiologist to review MR imaging studies in detail. These video sessions  provided real-time feedback, enabling continuous refinement of detection and segmentation accuracy and reinforcing key imaging principles.

The data scientists faced many challenges in detecting and segmenting rectal tumors, particularly in differentiating tumor boundaries from surrounding anatomical structures such as the rectal wall, bowel lumen, and adjacent organs. Tumors invading the rectal wall can blend seamlessly with normal tissue, while those extending into the lumen may be mistaken for stool or imaging artifacts. Additionally, the variability in imaging quality, patient anatomy, and the integration of multiple MR imaging sequences (e.g., T2- and diffusion-weighted MR images) add complexity to the segmentation process. Early attempts by data scientists frequently resulted in over-segmentation, where normal tissue or artifacts were included as part of the tumor. After corrective feedback, the data scientists tended to under-segment, omitting portions of infiltrative tumors. This iterative process highlighted the need for calibration to achieve an accurate balance.

Mentorship from a fellowship-trained radiologist was essential to address these challenges. The radiologist provided targeted guidance on identifying subtle imaging features, such as irregular signal intensities or loss of rectal wall integrity, to refine tumor delineation. Through regular feedback sessions, radiologists reviewed segmentation results, explained common errors, and demonstrated proper techniques for distinguishing tumors from surrounding structures. Calibration exercises, where radiologists corrected both over- and under-segmented tumors, were particularly valuable in aligning the data scientists’ performance with clinical standards. By fostering a deeper understanding of anatomical nuances and imaging artifacts, mentorship significantly improved segmentation accuracy and prepared the team for future applications.

### Data collection

In preparation for MR imaging, patients with locally advanced rectal cancer (LARC) were administered a micro-enema, 50–150 mL of rectal gel based on tumor location, and 1 mg IV dose of glucagon to minimize peristalsis. These patients were part of the SHORT-FOX clinical trial at Stanford, which evaluates the efficacy of combining short-course radiotherapy with chemotherapy (FOLFOXIRI) to achieve higher rates of clinical complete response and facilitate organ preservation in rectal cancer patients. This trial aims to determine whether this approach can serve as an effective alternative to surgery while maintaining oncological outcomes and improving quality of life [11]. All MR images analyzed in this study were obtained pre-treatment as part of this trial.

Subsequently, the clinical data for these 37 patients were obtained from the STAnford Research Repository (STARR) tools [12] and MR images from the Research Information Center (RIC). For the detection and manual segmentation of rectal tumors, we used the Digital Imaging and Communications in Medicine (DICOM) files of both the T2- and diffusion-weighted MR images [13]. DICOM is a widely accepted standard for handling, storing, and transmitting medical imaging information, ensuring accurate analysis and seamless data sharing among researchers. The T2-weighted images provided detailed anatomical images essential for delineating rectal tumor boundaries, while the diffusion-weighted images were essential for confirming the presence of tumors due to their characteristic bright appearance in these images.

### Detection of rectal tumors

Epic Electronic Health Records (EHR) [14] was used to review each patient’s medical chart and collect all relevant clinical information. Simultaneously, diagnostic images from radiology were accessed through the external PACS application eUnity [15]. The study radiologist had previously interpreted these MR images and made precise annotations to identify the rectal tumors.

To accurately locate the tumors, the data scientists consulted the radiology report within eUnity to determine the specific MR imaging series and slice number where the tumors were marked by the radiologist. As not all radiologist annotations were on T2- or diffusion-weighted series, this step was essential to identify the correct tumor location in Slicer v5.7.0. The data scientists then reviewed the identified MR imaging series alongside the axial T2-weighted series in eUnity to initiate the segmentation process.

The data scientists utilized a triangulation tool to cross-reference the radiologist’s annotations, enabling them to locate the corresponding slice in the axial T2-weighted images where the tumor was present. This approach ensured precise alignment and accurate detection of the tumor across different imaging series. They also examined the rectal tumor in the diffusion-weighted images to collect additional data. This process of cross-referencing and verifying the tumor location across multiple imaging series was essential to the accuracy of the data scientists’ segmentation efforts, ensuring that the rectal tumors were correctly identified and segmented before being reviewed by the radiologist.

### Manual segmentation of rectal tumors

The rectal tumors were manually segmented in 3D by two data scientists using Slicer v5.7.0. Among the 37 patients, 18 (48.6%) were segmented by one data scientist, while the remaining 19 (51.4%) were segmented by the other. For segmentation, DICOM files from the axial T2-weighted images  were imported into Slicer v5.7.0, with the slice numbers referenced in eUnity used to accurately locate the tumor. This method helped the data scientists detect the tumor within Slicer v5.7.0.

In the axial view, the selected region was adjusted to enhance contrast within the rectum, making the tumor more distinguishable. For small tumors, the pencil tool in the segment editor module was used to manually outline the tumor in each axial slice. For larger tumors, the paintbrush tool in the segment editor was employed, allowing the tumor to be painted with a round brush of varying sizes across the axial slices.

Following segmentation, the sagittal and coronal slices, as well as the 3D volume of the tumor, were thoroughly reviewed to ensure continuity and accuracy. This review process also included examining the diffusion-weighted images to confirm that the tumor, appearing bright, was correctly identified and segmented. Once the segmentation was verified, the tumor segmentation data was saved as a Nearly Raw Raster Data (NRRD) file, ready for radiologist review.

### Review

The study radiologist reviewed all 37 MR images along with their manual tumor segmentations in Slicer v5.7.0. A detailed spreadsheet was created and maintained to document the manual segmentations delineated by the data scientists, including any comments and observations. This spreadsheet was then shared with the radiologist. For each segmentation, the radiologist either made direct edits or provided detailed feedback on how the edits should be made. These edits were carefully documented in the spreadsheet, specifying the exact modifications made. This comprehensive log served as a reference for all T2-weighted images, the manual segmentations by the data scientists, and the corresponding edits and comments provided by the radiologist.

### Evaluation

To quantitatively evaluate the accuracy of the segmentations performed by the data scientists against the gold standard established by the radiologist, the Dice Similarity Coefficient (DSC) and Jaccard Index (JI) were used.. These metrics are widely recognized in the field of medical image analysis for their effectiveness in assessing segmentation quality. The DSC and JI are both metrics that range from 0 to 1, with 0 indicating no overlap between the segmentations and 1 representing perfect similarity, meaning the segmentations completely overlap. The DSC is often preferred because it provides a robust measure of similarity that is sensitive to the size of the overlapping regions between the predicted and reference segmentations. The JI, on the other hand, is a stricter measure as it penalizes discrepancies more severely [3, 4].

### Image pre-processing

**Fig. 2:**
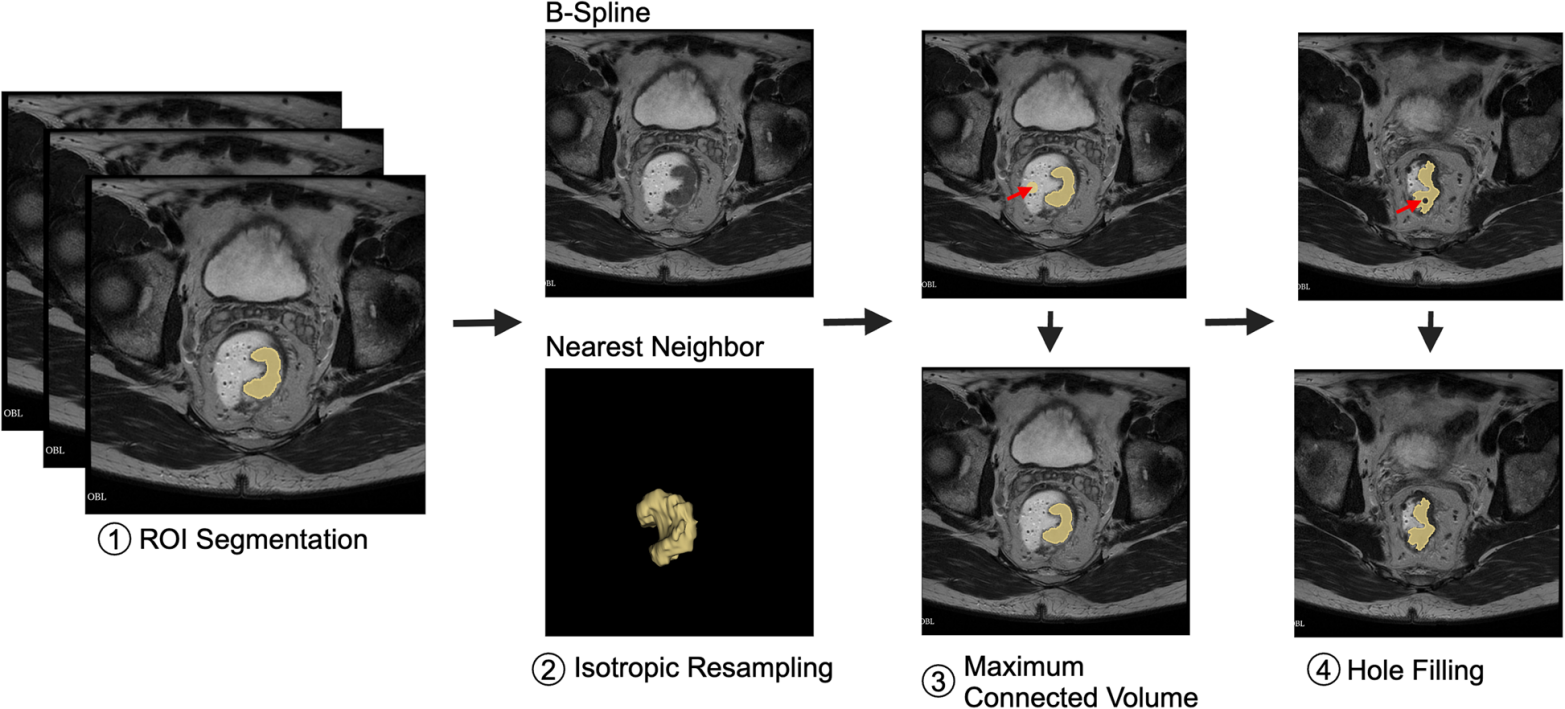
Overview of image pre-processing steps for data harmonization, including (1) ROI segmentation (yellow) delineated by data scientist and reviewed by radiologist, (2) isotropic resampling of MR images to a uniform spacing of 1 mm in the x, y, and z planes using B-Spline interpolation to preserve anatomical details, while segmentations were resampled using Nearest Neighbor (NN) interpolation to maintain boundary precision, balancing anatomical accuracy with segmentation fidelity, (3) extraction of the maximum connected component to remove stray pixels (denoted by red arrow), and (4) hole filling. Created in BioRender. Selby, H. (2025) https://BioRender.com/e41a602

Image pre-processing (Fig. 2) is crucial for data harmonization. In this study, MR images were spatially resampled to an isotropic spacing of 1 mm in the x, y, and z planes using B-Spline interpolation, which preserves anatomical details. Segmentations delineated by data scientist and reviewed by radiologist (yellow) were resampled using Nearest Neighbor (NN) interpolation to maintain the precision of tumor boundaries. This combination of B-Spline interpolation for imaging and NN interpolation for segmentations effectively balances the preservation of anatomical accuracy with boundary fidelity [16, 17].

Pre-processing also involved maximum connected volume and hole filling [18]. Maximum connected volume selection groups all connected voxels and, if there is more than one group, eliminates all but the group with the largest volume. Maximum connected volume selection ensures that the segmentation is focused solely on the tumor, reducing the likelihood of including smaller, irrelevant segments caused by stray pixels. Hole filling finds unsegmented regions that are completely surrounded by segmented regions and includes them in the segmentation to be processed. Hole filling identifies these holes and integrates them into the main segmentation, ensuring a more complete and accurate representation of the tumor.

By incorporating resampling, maximum connected volume selection, and hole filling, the segmentation process becomes more robust, resulting in a clearer and more reliable delineation of tumor boundaries. All image pre-processing was done in Python v3.13.0 using SimpleITK [19–21].

## Results

### Data collection

The clinical characteristics of the study participants are summarized in Table 1. The median age was 52 years [IQR: 44–61], with 35.14% women (*n=*13) and 64.86% men (*n=*24). All tumors included in this study were histologically confirmed as adenocarcinomas, with no cases of mucinous adenocarcinoma identified. Tumor locations were primarily in the lower rectum (43.24%, *n=*16) and mid-rectum (40.54%, *n=*15), with fewer cases in the high rectum (16.22%, *n=*6). Regarding tumor staging, the majority of participants were classified as T3 (70.27%, *n=*26), followed by T4 (21.62%, *n=*8) and T2 (8.10%, *n=*3). For nodal staging, 40.54% were N1 (*n=*15), another 40.54% were N2 (*n=*15), and 18.92% were N0 (*n=*7).

**Table 1:** Clinical characteristics

**Clinical Characteristic**		n (%)
Total Number of Patients		37 (100)
Age (y)^a^		52 [44–61]
Sex	Female	13 (35.14)
	Male	24 (64.86)
Histology	Adenocarcinoma	37
Tumor Location	Low	16 (43.24)
	Mid	15 (40.54)
	High	6 (16.22)
Tumor Stage	T2	3 (8.10)
	T3	26 (70.27)
	T4	8 (21.62)
Nodal Stage	N0	7 (18.92)
	N1	15 (40.54)
	N2	15 (40.54)

Data are presented as n (number of patients), with percentages in parentheses

^a^Medians are reported with inter-quartile ranges (IQR) in square brackets

Table 2 summarizes the MR imaging characteristics for the study participants. All participants (*n=*37) underwent T2- and diffusion-weighted imaging. Among the T2-weighted images, the majority were acquired using General Electric scanners (64.86%, *n*=24), followed by Siemens (21.6%, *n=*8) and Philips (13.51%, *n=*5). The predominant slice thickness was 3 mm, comprising 91.89% (*n=*34) of images, with smaller proportions at 2.5 mm (5.41%, *n=*2) and 3.5 mm (2.70%, *n=*1).

**Table 2:** MR imaging characteristics

MR Imaging Characteristic		n (%)
T2-weighted Images		37 (100)
Manufacturer	General Electric	24 (64.86)
	Siemens	8 (21.62)
	Philips	5 (13.51)
Slice Thickness (mm)	2.5	2 (5.41)
	3	34 (91.89)
	3.5	1 (2.70)
Diffusion-weighted Images		106 (100)
Manufacturer	General Electric	70 (66.04)
	Siemens	21 (19.81)
	Philips	15 (14.15)
Slice Thickness (mm)	2.7	3 (2.83)
	3	81 (76.42)
	4	3 (2.83)
	4.2	3 (2.83)
	5	11 (10.38)
	7	3 (2.83)
	8	2 (1.89)
Diffusion B-value	0	34 (32.08%)
	50	29 (27.36)
	100	2 (1.89)
	400	3 (2.83)
	600	1 (0.94)
	800	24 (22.64)
	1000	9 (8.49)
	1400	4 (3.77)

Data are presented as n (number of MR images), with percentages in parentheses

For diffusion-weighted images, General Electric scanners were also the most commonly used 66.04% (*n=*70), with Siemens and Philips contributing 19.81% (*n=*21) and 14.15% (*n=*15) respectively. Slice thickness varied more widely for DWI, with 3 mm being the most frequent (76.42%, *n=*81). Other slice thicknesses included 5 mm (10.38%, *n=*11), 2.7 mm, 4 mm, 4.2 mm, 7 mm (each 2.83%, *n=*3), and 8 mm (1.89%, *n=*2).The diffusion B-value distribution showed the highest frequency at 0 (32.08%, *n=*34), followed by 50 (27.36%, *n=*29), and 800 (22.64%, *n=*24). Other diffusion B-values included 100 (1.89%, *n=*2), 400 (2.83%, *n=*3), 600 (0.94%, *n=*1), 1000 (8.49%, *n=*9), and 1400 (3.77%, *n=*4). These MR imaging characteristics highlight the consistency and diversity of acquisition parameters used in this study.

### Detection of rectal tumors

With the help of radiologist annotations and radiology notes in the Epic EHR, the data scientists successfully detected all of the tumors on both T2- and diffusion-weighted MR images. This outcome suggests that, with radiologist guidance, annotations, and clinical information, data scientists can assist in tumor detection across multiple MR imaging modalities.

### Manual segmentation of rectal tumors

**Fig. 3:**
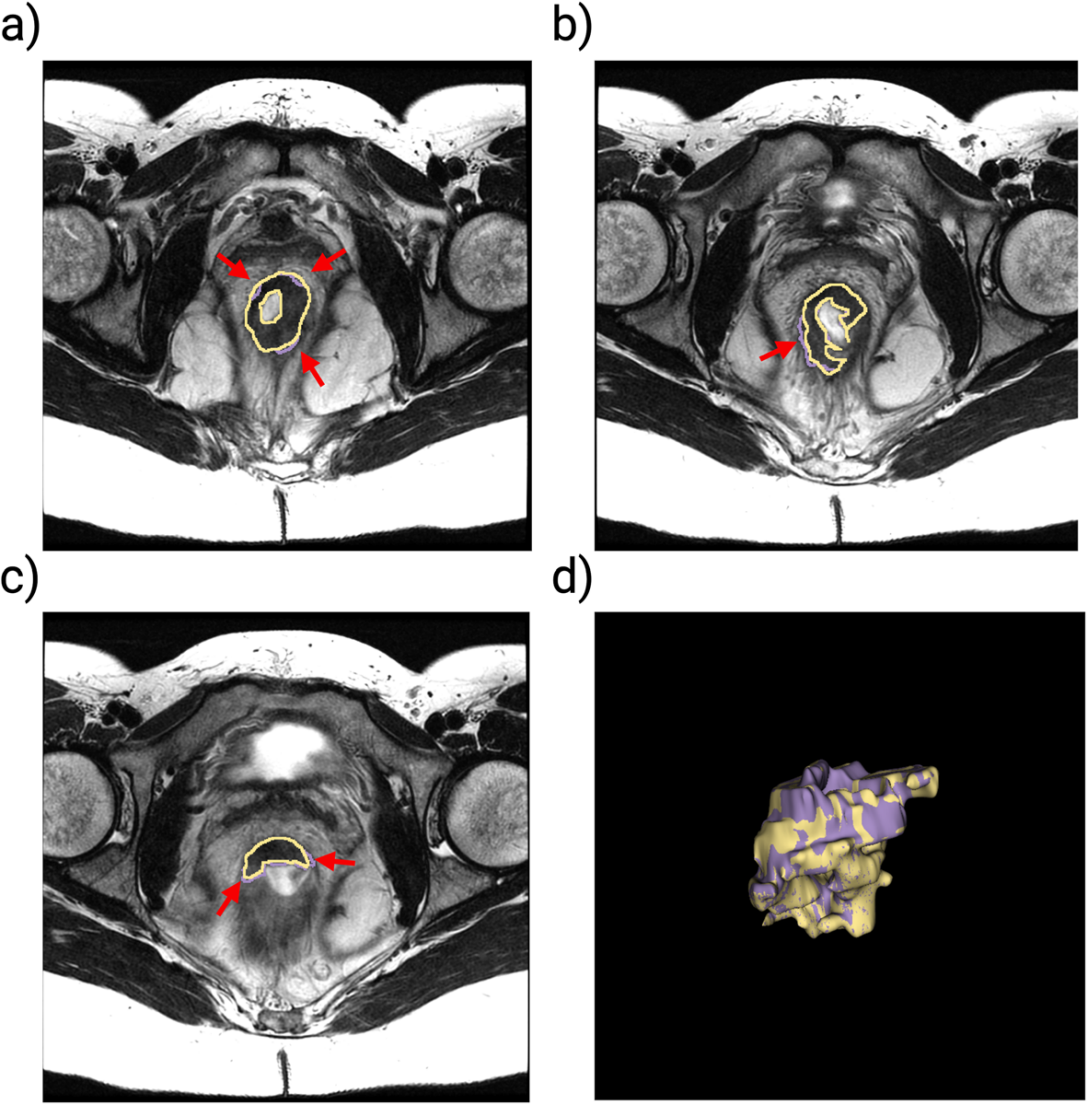
Selected axial T2-weighted MR slices from a patient (SFX-024 in Table 3) with T3N0 rectal cancer, highlighting challenging segmentation cases. The MR images are overlaid with tumor segmentations that were (a) over-segmented, (b) under-segmented, and (c) over-segmented by the data scientist (yellow) with subsequent edits by the radiologist (purple). Image (d) presents a 3D visualization of the segmented tumor, illustrating the spatial relationship and overlap between the data scientist’s segmentation (yellow) and the radiologist’s edits (purple). The DSC and JI between the segmentations performed by the data scientist and the radiologist were 0.98 and 0.96, respectively. Created in BioRender. Selby, H. (2025) https://BioRender.com/t87e418

Figure 3 provides an exemplar visual representation of a challenging tumor segmentation by a data scientist (yellow), and edited by radiologist (purple) for a patient diagnosed with T3N0 rectal cancer, with a tumor located in the lower rectum. This figure illustrates the differences and similarities between the segmentation performed by a data scientist and a radiologist.

Figure 3 presents selected axial T2-weighted MR slices from a patient (SFX-024 in Table 3) with T3N0 rectal cancer, illustrating the challenges in tumor segmentation and the differences between the initial delineations by the data scientist and the corrections made by the radiologist. In MR images (a) and (c), the tumor is initially over-segmented by the data scientist and subsequently corrected by the radiologist. In image (b), the tumor is under-segmented by the data scientist and similarly corrected by the radiologist. The radiologist noted that these segmentations were particularly challenging and required reference to additional imaging sequences. For the distal left tumor wall, the radiologist determined that some regions initially included in the segmentation were normal tissue rather than tumor. Image (d) presents a 3D VOI with segmentations delineated by the data scientist and radiologist overlaid, offering a direct visual comparison that emphasizes areas of agreement and discrepancy between their approaches. These 3D visualizations are essential to understand how segmentation decisions in 2D slices translate into 3D models, which is particularly important in a clinical context where precise volumetric assessments play a key role in treatment planning and prognosis. The DSC and JI between the data scientists’ and radiologist’s segmentations were 0.98 and 0.96, respectively, demonstrating a high level of agreement despite the challenging nature of the case.

**Table 3:** Data Scientist VOI (mm^3^), Radiologist VOI (mm^3^), Dice Similarity Coefficient (DSC), and Jaccard Index (JI) comparing rectal tumor segmentations delineated by data scientists and the radiologist

Patient ID	Data Scientist VOI (mm^3^)	Radiologist VOI (mm^3^)	Segmentation Error	DSC	JI
SFX-001	15596.30	14323.88	Over	0.89	0.80
SFX-006	31178.41	23466.99	Over	0.71	0.55
SFX-009	15698.44	13358.21	Over	0.90	0.81
SFX-010	30195.28	23436.80	Over	0.81	0.68
SFX-012	33292.50	18925.50	Over	0.68	0.52
SFX-024	15061.69	15209.08	Under	0.98	0.96
SFX-032	10325.10	11641.12	Under	0.94	0.88
SFX-033	24884.48	24758.01	Over	0.83	0.71
SFX-036	18109.10	18506.85	Under	0.98	0.96

### Review

The radiologist conducted a comprehensive review of all 37 T2 and 106 diffusion-weighted MR images, including the segmentations delineated by the two data scientists. In this process, the radiologist identified discrepancies in 9 out of the 37 segmentations (24.32%), requiring corrections to ensure alignment with clinical standards. This review highlights the importance of expert involvement in ensuring the accuracy of tumor segmentations.

### Evaluation

The rectal tumor segmentations delineated by the data scientists and edited by the radiologist showed a high level of agreement, with a mean DSC [95% CI] of 0.965 [0.939–0.992] and a mean JI [95% CI] of 0.943 [0.900–0.985], as summarized in Table 3. Note that the mean [95% CI] values include all 37 segmentations in the study, not just the 9 edited cases listed in Table 3. These metrics indicate that the segmentations had minimal variance between the two approaches. Discrepancies in cases with slightly lower DSC and JI values were primarily due to over- or under-segmentation by the data scientists.

From the data in Table 3, there are 6 cases of over-segmentation and 3 cases of under-segmentation. Over-segmentation errors were identified when the VOI delineated by the data scientist was larger than the VOI segmented by the radiologist (Data Scientist VOI $\ge$ Radiologist VOI). Conversely, under-segmentation errors occurred when the radiologist’s VOI was greater than the data scientist’s VOI (Radiologist VOI > Data Scientist VOI).

For instance, over-segmentation errors often resulted from extending segmentation across too many images along the z-axis, thereby overestimating the tumor’s extent. Conversely, under-segmentation errors arose when portions of infiltrative tumors with subtle invasion into the rectal wall or adjacent structures were omitted, leading to an underestimation of the tumor’s true size. Such errors typically occurred in regions with low contrast or signal variability, where tumor boundaries were less distinct. These discrepancies were identified and corrected by the radiologist to ensure accurate delineation.

Despite these adjustments, the strong agreement demonstrated in Table 3 suggests that data scientist-generated segmentations – informed by radiologist annotations, radiology notes in Epic EHR, radiologist-led training, and CRC MDTB discussions – can approach the accuracy of an experienced radiologist when performed with radiologist guidance. This finding underscores the potential for collaborative approaches in developing AI-assisted segmentation tools, ultimately aiming to enhance efficiency and reproducibility in clinical workflows.

### Image pre-processing

The pre-processing steps harmonized the MR images and segmentations, ensuring accurate tumor delineation. B-Spline interpolation was used to resample MR images to an isotropic 1 mm spacing, preserving fine anatomical details and minimizing artifacts. For tumor segmentations, NN interpolation was applied to maintain the discrete nature of labels and ensure sharp, precise boundaries. This combination balances anatomical accuracy with boundary fidelity, ensuring reliable segmentation and robust data for clinical and research applications. Maximum connected volume selection was utilized to eliminate small, irrelevant segments. Among the 37 segmentations, two segmentations were found to contain stray pixels, which were effectively removed during this process. Additionally, 11 segmentation holes were identified and filled, resulting in more complete and accurate tumor delineations. Image pre-processing enhanced the uniformity and accuracy of the segmentations, ensuring more reliable tumor delineations for subsequent analyses.

## Discussion

In this study, two data scientists worked alongside a radiologist to detect and segment rectal tumors on T2-weighted MR imaging. This collaborative effort aimed to streamline manual segmentation while also generating high-quality segmentations to train an AI-driven automated segmentation model. To prepare for this task, the data scientists underwent radiologist-led training, participated in hands-on practice with real-time feedback from a radiologist, and attended Stanford’s weekly CRC MDTB to gain clinical insights. The data scientists leveraged clinical information and radiologist-annotated MR images from the Epic EHR to accurately identify rectal tumors in Slicer v5.7.0 for 37 patients. Segmentations were performed on the T2-weighted images using Slicer v5.7.0, and all MR images and their tumor segmentations delineated by the data scientists were subsequently reviewed and edited by the radiologist. Minor corrections were needed in 9 of 37 segmentations, while the remaining segmentations required no adjustments demonstrating alignment with the radiologist’s segmentations. The high concordance in tumor segmentation between the data scientists and the radiologist, as reflected by a mean DSC [95% CI] of 0.965 [0.939–0.992] and a mean JI [95% CI] of 0.943 [0.900–0.985], suggests a high degree of overlap, though some variability remained.

The use of hydrophilic gel for rectal distension is controversial, though part of the standard imaging protocol at our institution. While rectal distension may influence segmentation accuracy by distorting the cleavage planes between the tumor and adjacent structures, it has also been shown to improve variability in rectal delineation between observers [22]. Although the gel helped enhance the visibility of the rectum and its surrounding structures, such distension could have contributed to segmentation challenges, especially in areas where tumor boundaries were close to adjacent tissues. Previous study indicates that rectal filling dose not alter the distance to the mesorectal fascia but can increase the distance from the anal sphincter complex [23]. Additionally, rectal distension may introduce artefactual distortions in diffusion-weighted imaging, which could impact segmentation accuracy. While these distortions were minimal in this study, they remain a potential source of error in cases with higher variability. Furthermore, although no mucinous adenocarcinoma was present in this cohort, T2-hyperintensity typically associated with such tumors is known to complicate segmentation and could lead to errors in studies involving this histological subtype. Future studies with larger, more diverse datasets are needed to evaluate the extent of these challenges in broader contexts.

Unlike many other cancers, such as brain, breast, prostate, liver, or head and neck, there is no publicly available MR imaging dataset specifically for rectal cancer [10]. This lack of accessible rectal cancer MR imaging data poses a significant challenge for advancing automated segmentation techniques. To address this gap, we utilized a small-scale, high-quality, institution-specific dataset in this study. Given that tumor segmentation is labor-intensive, time-consuming, and costly, the radiologist provided guidance to data scientists on tumor detection and segmentation. This collaborative approach distributed the workload, saving time and reducing costs while maintaining high-quality segmentation accuracy.

Common challenges in tumor segmentation include segmentation errors, inter- and intra-reader variability, and limited integration with clinical workflows. In our study, we mitigated these issues by fostering collaboration between data scientists and the radiologist. Real-time feedback and iterative learning helped reduce segmentation errors, while PACS was used in the clinical setting and Slicer v5.7.0 in the research setting to bridge the gap between research workflows and clinical practice. However, for segmentation to be fully effective in clinical practice, integrating Slicer v5.7.0 directly into PACS would be essential.

This study has several limitations. First, the sample size was small, and the study was conducted at a single institution. While the MR images were high-quality and followed the same imaging protocol, the findings may not generalize to other institutions or imaging systems. Multi-center studies are needed to validate this workflow across diverse patient populations, imaging protocols, and MR systems to ensure broad applicability. Second, the review and editing of segmentations were performed by a single radiologist. While this ensured consistency in the review process, it may have introduced subjective bias, as the radiologist’s edits could have been influenced by their individual interpretation and the initial segmentations provided by the data scientists. Including a second and even third radiologist to independently perform manual segmentations would provide additional perspectives, reduce potential bias, and help quantify inter-reader variability. Future studies should incorporate multiple radiologists to compare segmentations, assess areas of disagreement, and validate the robustness of automated workflows. Furthermore, training AI models on the intersection or consensus of manual segmentations from multiple radiologists could improve model accuracy and generalizability by capturing the most reliable tumor boundaries. Third, although the radiologist reviewed and edited the segmentations delineated by the data scientists, there is a possibility that their edits were shaped by these segmentations. Automated segmentation could minimize such biases by providing objective, reproducible delineations that can serve as a baseline for validation by multiple radiologists. Lastly, while the collaborative approach effectively distributed workload, radiologist involvement was still required for quality assurance. Automated segmentation could potentially reduce this need, streamlining the process and increasing efficiency.

## Conclusion

Our study suggests that, with radiologist training, oversight, and review, data scientists can support the detection and segmentation of rectal tumors on MR images, achieving a high degree of agreement with radiologist's segmentations. This collaborative effort enabled us to expand tumor detection and segmentation efforts for rectal cancer, a process that is labor-intensive, time-consuming, and costly when performed manually by a radiologist. By engaging data scientists in the detection and manual segmentation process, we were able to reduce some of the radiologist’s workload. However, radiologist review remains essential to ensure the accuracy of both the images and segmentations. This hands-on involvement also provided data scientists with a deeper understanding of rectal cancer, MR image interpretation, and the role of T2- and diffusion-weighted imaging, which is important as they will be responsible for pre-processing the data and training the AI models. With a curated training set that includes MR images and corresponding segmentations, we are now well-positioned to develop AI models capable of automating rectal tumor segmentation on MR images.

## Supplementary Information

Supplementary Material 1.

## Data Availability

The data utilized in this study contains protected health information (PHI) and is subject to HIPAA regulations, which prohibit public sharing to safeguard patient privacy. De-identified data may be made available upon reasonable request, subject to approval by the appropriate Institutional Review Board (IRB) or ethics committee, in accordance with HIPAA guidelines.
