# Supplementary material for: AI-ready rectal cancer MR imaging: a workflow for tumor detection and segmentation
Source: BMC Med Imaging. 2025 Mar 14;25:88. doi: 10.1186/s12880-025-01614-3 (PMC11909848; doi:10.1186/s12880-025-01614-3)
Supplement: Supplementary file 1 — Supplementary Material 1. [file 12880_2025_1614_MOESM1_ESM.pdf]

Supplemental Materials

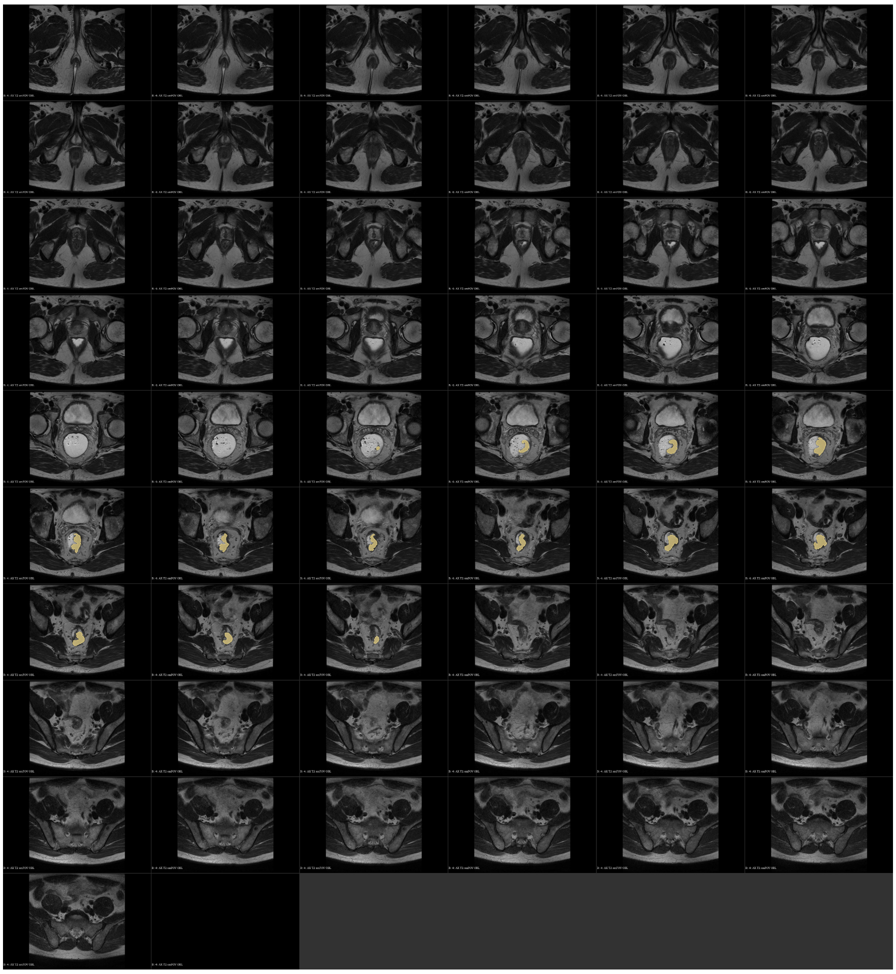

Fig. S1 Serial 2D z-slices extracted from the 3D MRI scan shown in Fig 2. Each slice provides a distinct axial view, highlighting the tumor and surrounding anatomical structures. The rectal tumor segmented by the data scientist is indicated in yellow.

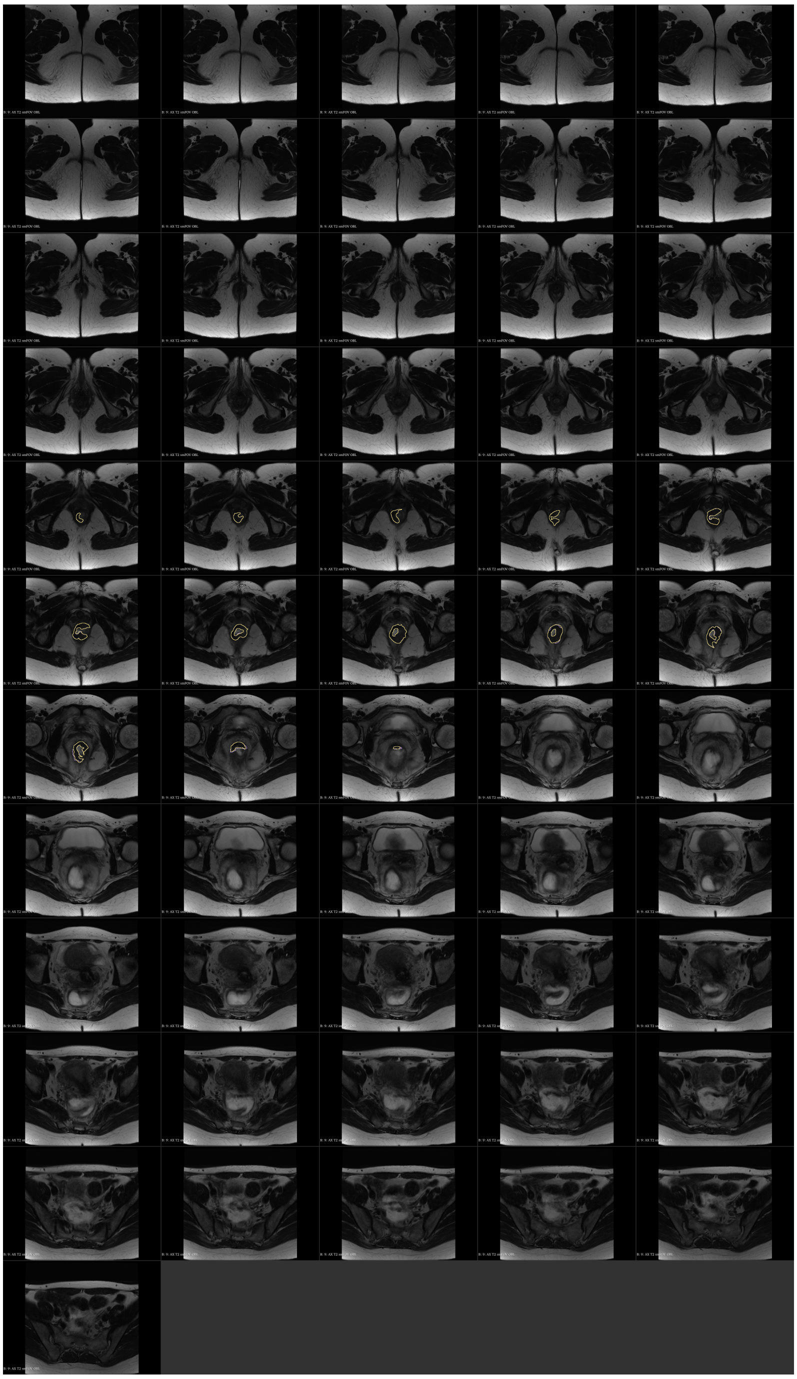

Fig. S2 Serial 2D z-slices extracted from the 3D MRI scan presented in Fig 3. Each slice illustrates a distinct axial view, highlighting the tumor and surrounding anatomical structures. The rectal tumor segmented by the data scientist is depicted in yellow, while the radiologist-refined segmentation is shown in purple.
